# Supplementary material for: Cross-modal autoencoder framework learns holistic representations of cardiovascular state
Source: Nat Commun. 2023 Apr 28;14:2436. doi: 10.1038/s41467-023-38125-0 (PMC10140057; doi:10.1038/s41467-023-38125-0)
Supplement: Supplementary file 1 — Supplementary Information [file 41467_2023_38125_MOESM1_ESM.pdf]

Supplementary Materials: Cross-Modal Autoencoder Framework Learns Holistic Representations of Cardiovascular State

Adityanarayanan Radhakrishnan[1#], Sam F. Friedman[2#], Shaan Khurshid[2,3], Kenney Ng[4], Puneet Batra[2], Steven A. Lubitz[2,3*], Anthony A. Philippakis[2,*], Caroline Uhler[1,2,*]

[1]Massachusetts Institute of Technology, U.S.A.
[2]Broad Institute of MIT and Harvard, U.S.A.
[3]Massachusetts General Hospital, U.S.A.
[4]IBM T.J. Watson Research Center, U.S.A.

[#]Equal contribution.
[*]To whom correspondence should be addressed; E-mail: cuhler@mit.edu; aphilipp@broadinstitute.org; lubitz@broadinstitute.org.

This PDF file includes:

Supplementary Figures S1 to S17
Supplementary Tables S1 to S3
Supplementary Videos S1 to S5

Supplementary Figures

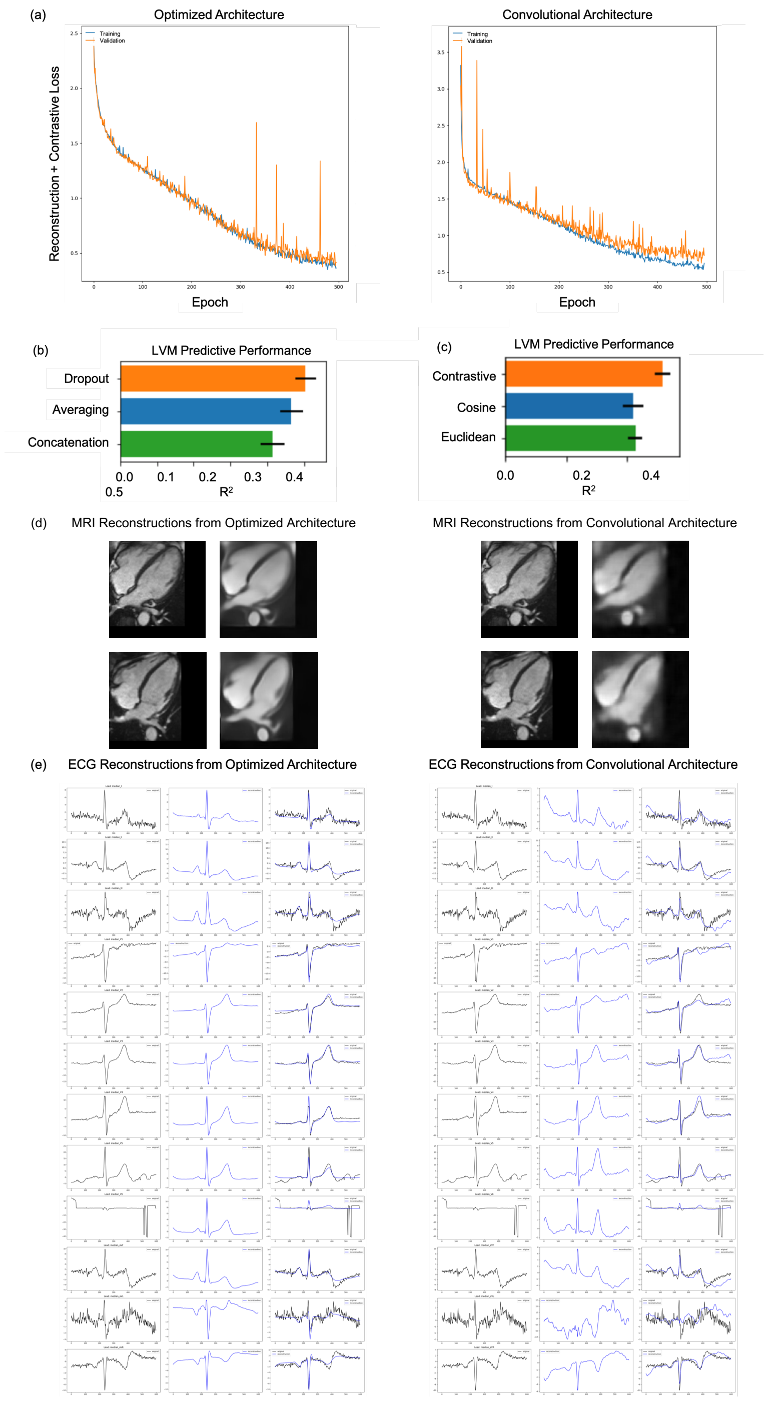

Supplementary Fig. S1: The impact of hyper-parameters, architecture, and loss functions on the performance of our autoencoder. (a) We visualize the training and validation loss across 500 epochs; the hyper-parameter optimized architecture produces lower loss than the standard convolutional network architecture from [1]. (b) Using modality dropout leads to improved performance on downstream LVM prediction over averaging or concatenating modality-specific embeddings during training (n=4708, bar shows mean value and black line indicates +/- one standard deviation). (c) Contrastive loss to pair modalities in the latent space leads to improved performance on LVM prediction over pairing samples by maximizing cosine similarity or minimizing Euclidean distance (n=4708, bar shows mean value and black line indicates +/- standard deviation). (d-e) We visualize the reconstructions of test MRI and ECG samples from the optimized architecture and standard convolutional architecture; reconstructions from the optimized architecture are of higher quality (lower loss) than those from the standard convolutional architecture.

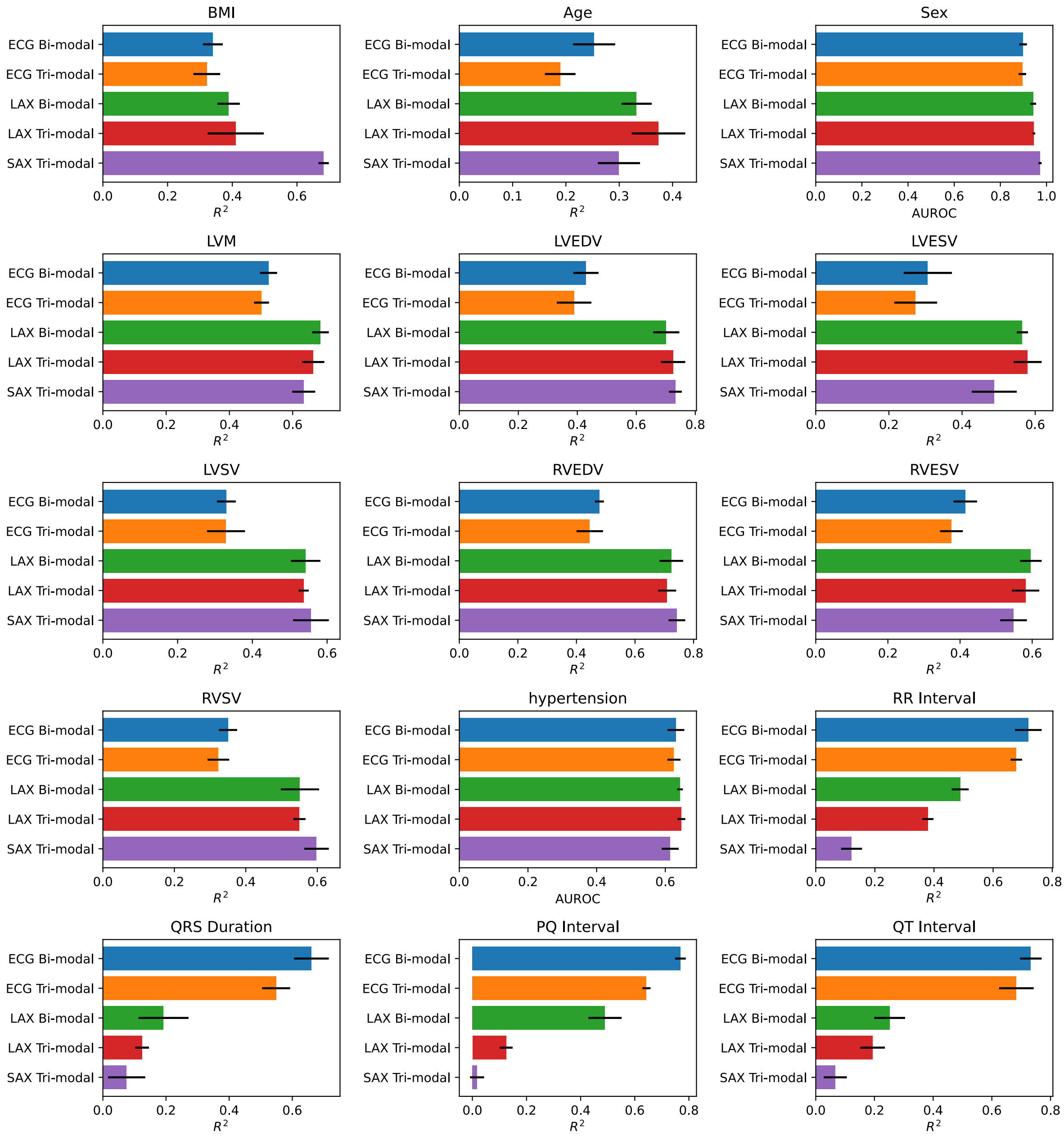

Supplementary Fig. S2: A comparison of phenotype prediction from bi-modal embeddings of ECGs and long axis views of cardiac MRIs (LAX) and tri-modal embeddings of ECGs, LAX, and short axis view of cardiac MRIs (SAX). We observe that predictive performance of general phenotypes such as BMI and sex improves when using tri-modal embeddings since SAX is informative about these phenotypes. On the other hand, prediction of ECG intervals (e.g., QT, RR, and PQ intervals) from tri-modal embeddings decreases since SAX contains little information about these phenotypes and may add noise to the prediction task. For BMI and Age, $n = 4212$. For Sex and hypertension, $n = 4218$. For LVM, LVEDV, LVESV, LVSV, RVEDV, RVESV, and RVSV, $n = 4218$. For RR Interval, QRS Duration, PQ Interval and QT Interval, $n = 4120$. Bars shows mean value and black line indicates +/- standard deviation.

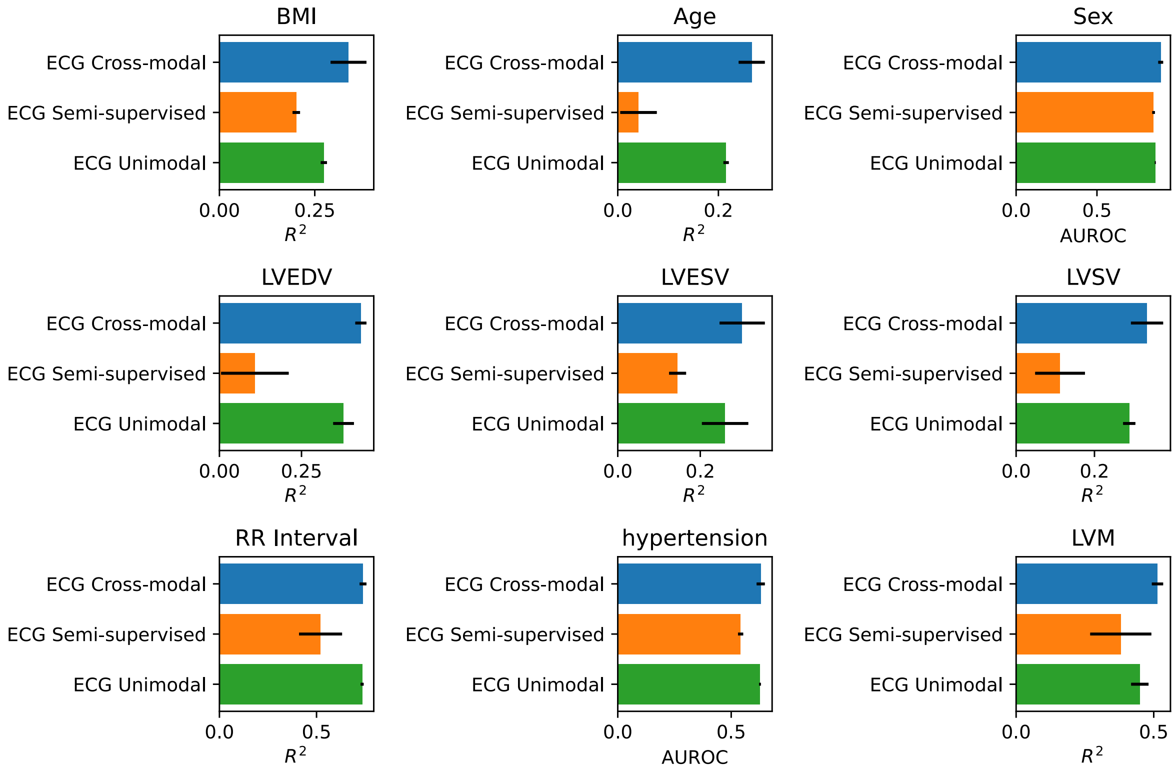

Supplementary Fig. S3: Prediction from cross-modal ECG embedding outperforms prediction from unimodal ECG embedding and semi-supervised ECG embedding, i.e., the embedding obtained from an autoencoder that is trained to both reconstruct ECGs and predict phenotypes. For BMI and Age, $n = 4212$. For Sex and hypertension, $n = 4218$. For LVEDV, LVESV, LVSV, and LVM, $n = 4218$. For RR Interval, $n = 4120$. Bars shows mean value and black line indicates +/- standard deviation.

(a) R^2 Values for ECG-Derived Phenotype Prediction from Cross-modal MRI Embeddings

Model\Phenotype	PQ Interval	QT Interval	QTC Interval	QRS Duration	RR Interval	Average
Kernel Regression	0.51	0.26	0.19	0.25	0.49	0.34
Linear Regression	0.50	0.25	0.18	0.24	0.49	0.33

R^2 Values for MRI-Derived Phenotype Prediction from Cross-modal ECG Embeddings

Model\Phenotype	LVM	LVEDV	LVEF	LVESV	LVSV	RVEF	RVESV	RVSV	RVEDV	Average
Kernel Regression	0.53	0.45	0.11	0.39	0.31	0.13	0.44	0.31	0.48	0.35
Linear Regression	0.51	0.43	0.11	0.36	0.30	0.14	0.43	0.30	0.47	0.34

(b) R^2 Values for General Numerical Phenotype Prediction from Cross-modal ECG Embeddings

Model\Phenotype	BMI	Age	Average
Kernel Regression	0.36	0.27	0.32
Linear Regression	0.35	0.24	0.29

R^2 Values for General Numerical Phenotype Prediction from Cross-modal MRI Embeddings

Model\Phenotype	BMI	Age	Average
Kernel Regression	0.48	0.42	0.45
Linear Regression	0.47	0.40	0.44

(c) AUROC Values for General Categorical Phenotype Prediction from Cross-modal ECG Embeddings

Model\Phenotype	Sex	Hypercholesterolemia	Hypertension	Average
Kernel Regression	0.96	0.64	0.69	0.76
Logistic Regression	0.90	0.56	0.63	0.70

AUROC Values for General Categorical Phenotype Prediction from Cross-modal MRI Embeddings

Model\Phenotype	Sex	Hypercholesterolemia	Hypertension	Average
Kernel Regression	0.99	0.66	0.75	0.80
Logistic Regression	0.95	0.58	0.66	0.73

Higher R^2 and AUROC are better with a maximum value of 1.

Supplementary Fig. S4: Comparison of kernel, linear, and logistic regression models used for phenotype prediction from cross-modal ECG and MRI embeddings. Overall, the kernel regression models outperform linear and logistic regression models for the tasks considered in Fig. 2 of the main text. (a, b) R^2-values for kernel and linear regression used in the prediction of continuous-valued phenotypes considered in Fig. 2 of the main text. (c) Area under the Receiver Operating Curve (AUROC) for kernel and logistic regression used in the prediction of categorical phenotypes considered in Fig. 2 of the main text.

AUROC Values for Left Ventricular Hypertrophy Classification

	Cross-modal ECG	Unimodal ECG	Supervised ECG
LVH AUROC	**0.756 ± 0.022**	0.716 ± 0.012	0.692 ± 0.016
LVSD AUROC	**0.572 ± 0.052**	0.535 ± 0.028	0.558 ± 0.023

Supplementary Fig. S5: Logistic regression using cross-modal ECG embeddings leads to improved prediction of Left Ventricular Hypertrophy (LVH) and Left Ventricular Systolic Dysfunction (LVSD) over logistic regression from unimodal ECG embeddings and supervised learning from ECGs directly.

Prediction of MRI Derived Phenotypes from ECG (R^2)

	Without LDL & CRP			With LDL & CRP		
	Cross-modal	Unimodal	Supervised	Cross-modal	Unimodal	Supervised
LVM	**0.536**	0.475	0.439	**0.536**	0.485	0.440
LVEDV	**0.451**	0.382	0.381	0.442	0.379	0.383
LVEF	**0.103**	0.080	0.049	0.098	0.085	0.044
LVESV	**0.380**	0.324	0.327	0.368	0.325	0.326
LVSV	**0.316**	0.246	0.231	0.315	0.244	0.233
RVEF	**0.129**	0.116	0.065	0.129	0.120	0.063
RVESV	0.445	0.388	0.374	**0.447**	0.399	0.380
RVSV	**0.320**	0.245	0.236	**0.320**	0.248	0.239
RVEDV	**0.490**	0.409	0.407	**0.490**	0.418	0.413
Average	**0.352**	0.296	0.279	0.349	0.300	0.280

Prediction of General Phenotypes from ECG (R^2)

	Without LDL & CRP			With LDL & CRP		
	Cross-modal	Unimodal	Supervised	Cross-modal	Unimodal	Supervised
BMI	0.362	0.320	0.192	**0.461**	0.426	0.330
Age	0.264	0.253	0.105	**0.278**	0.253	0.117
Average	0.313	0.286	0.148	**0.370**	0.340	0.224

Prediction of General Phenotypes from ECG (AUROC)

	Without LDL & CRP			With LDL & CRP		
	Cross-modal	Unimodal	Supervised	Cross-modal	Unimodal	Supervised
Sex	0.961	0.937	0.911	**0.962**	0.947	0.909
Hypercholesterolemia	**0.635**	0.629	0.598	0.630	0.625	0.572
Hypertension	0.696	0.713	0.684	**0.706**	0.703	0.685
Average	0.764	0.760	0.731	**0.766**	0.758	0.722

Supplementary Fig. S6: Impact of two circulating biomarkers, namely low-density lipoprotein (LDL) and C-reactive protein (CRP), on the prediction of phenotypes from ECG. Generally, we observe that the inclusion of these biomarkers consistently increases the R^2-value for predicting BMI and age across all models but does not generally increase prediction accuracy for MRI derived phenotypes or sex, hypercholesterolemia, and hypertension.

Prediction of MRI Derived Phenotypes from ECG (R^2)

	Without Sex			With Sex		
	Cross-modal	Unimodal	Supervised	Cross-modal	Unimodal	Supervised
LVM	0.535	0.469	0.433	**0.594**	0.576	0.490
LVEDV	0.452	0.387	0.391	**0.506**	0.495	0.415
LVEF	0.109	0.095	0.049	**0.123**	0.112	0.052
LVESV	0.388	0.344	0.352	**0.437**	0.428	0.369
LVSV	0.311	0.240	0.220	**0.345**	0.319	0.241
RVEF	0.131	0.111	0.061	**0.146**	0.140	0.073
RVESV	0.438	0.382	0.370	**0.493**	0.489	0.404
RVSV	0.312	0.242	0.222	**0.356**	0.337	0.246
RVEDV	0.479	0.403	0.399	**0.542**	0.530	0.436
Average	0.351	0.297	0.277	**0.394**	0.381	0.303

Prediction of MRI Derived Phenotypes from ECG (R^2)

	Without BMI			With BMI		
	Cross-modal	Unimodal	Supervised	Cross-modal	Unimodal	Supervised
LVM	0.535	0.469	0.433	**0.566**	0.513	0.475
LVEDV	0.452	0.388	0.391	**0.471**	0.428	0.400
LVEF	0.108	0.094	0.049	**0.111**	0.085	0.048
LVESV	0.388	0.344	0.352	**0.401**	0.359	0.346
LVSV	0.312	0.242	0.222	**0.328**	0.286	0.241
RVEF	0.131	0.113	0.061	**0.132**	0.099	0.066
RVESV	0.439	0.382	0.370	**0.445**	0.398	0.367
RVSV	0.313	0.244	0.223	**0.340**	0.296	0.250
RVEDV	0.480	0.403	0.400	**0.499**	0.446	0.409
Average	0.351	0.298	0.278	**0.366**	0.323	0.289

Supplementary Fig. S7: Impact of stratification by sex and BMI on the prediction of MRI-derived phenotypes from ECG. In general, we observe that such stratification increases the prediction accuracy as measured by R^2-values.

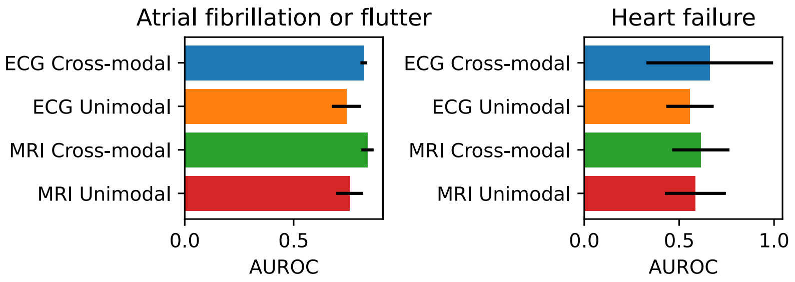

Supplementary Fig. S8: Cross-modal representation leads to improved performance of atrial fibrillation (AF) and heart failure (HF). Labels of AF and HF were provided by the UK Biobank. For these phenotypes, $n = 4708$ and bars shows mean value and black line indicates +/- standard deviation.

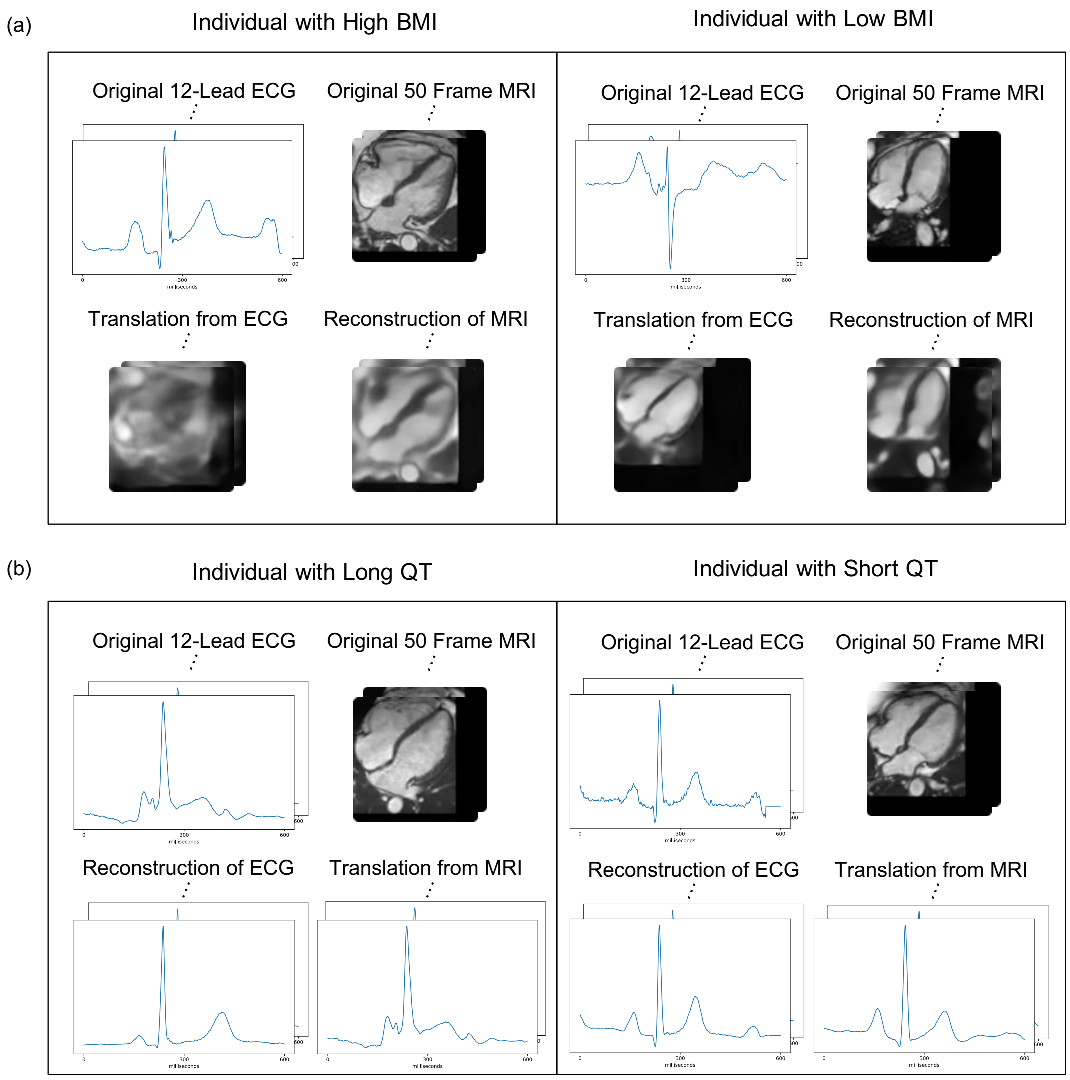

Supplementary Fig. S9: Additional examples of modality translation using cross-modal autoencoders. (a) Translation of ECG to MRI for individuals with high and low BMI. (b) Translation of MRI to ECG for individuals with long and short QT intervals.

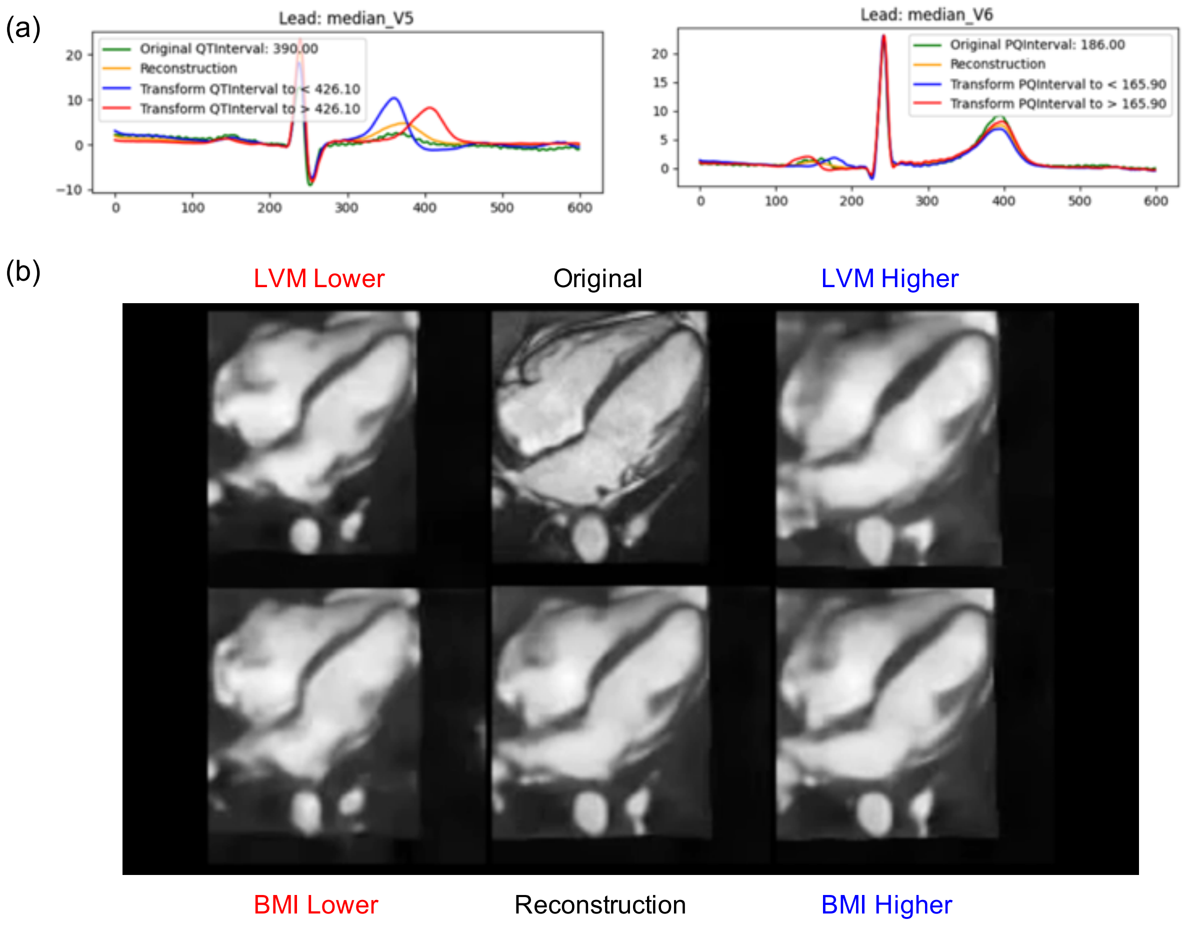

Supplementary Fig. S10: Translating cross-modal embeddings along a phenotype direction produces phenotype-specific impacts on ECGs and MRIs after decoding. (a) Translating cross-modal embeddings along the direction from short to long (or long to short) QT or PQ interval leads to corresponding increases (or decreases) of these intervals on the original ECGs. (b) Translating cross-modal embeddings along the direction from low to high (or high to low) LVM or BMI leads to corresponding increases (or decreases) of these phenotypes on the original MRI.

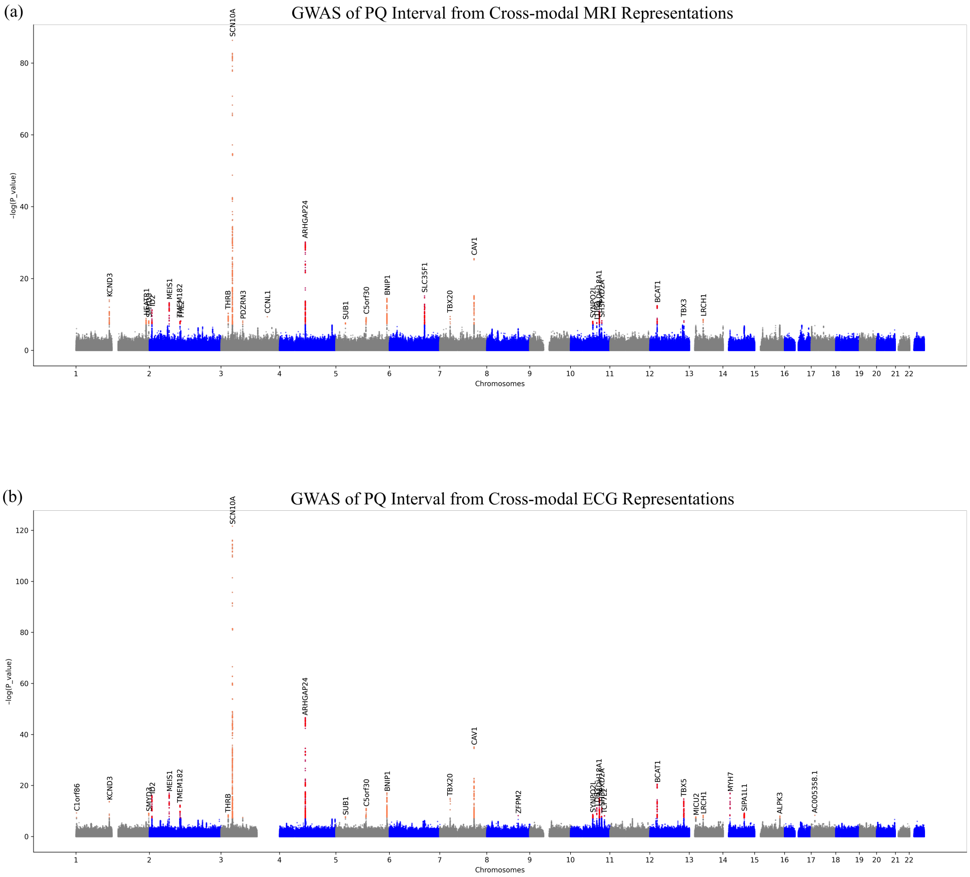

Supplementary Fig. S11: GWAS of PQ interval predicted from (a) MRI cross-modal representations and (b) ECG cross-modal representations identify genes associated with PQ interval duration, including SCN10A, KCND3, and CAV1.

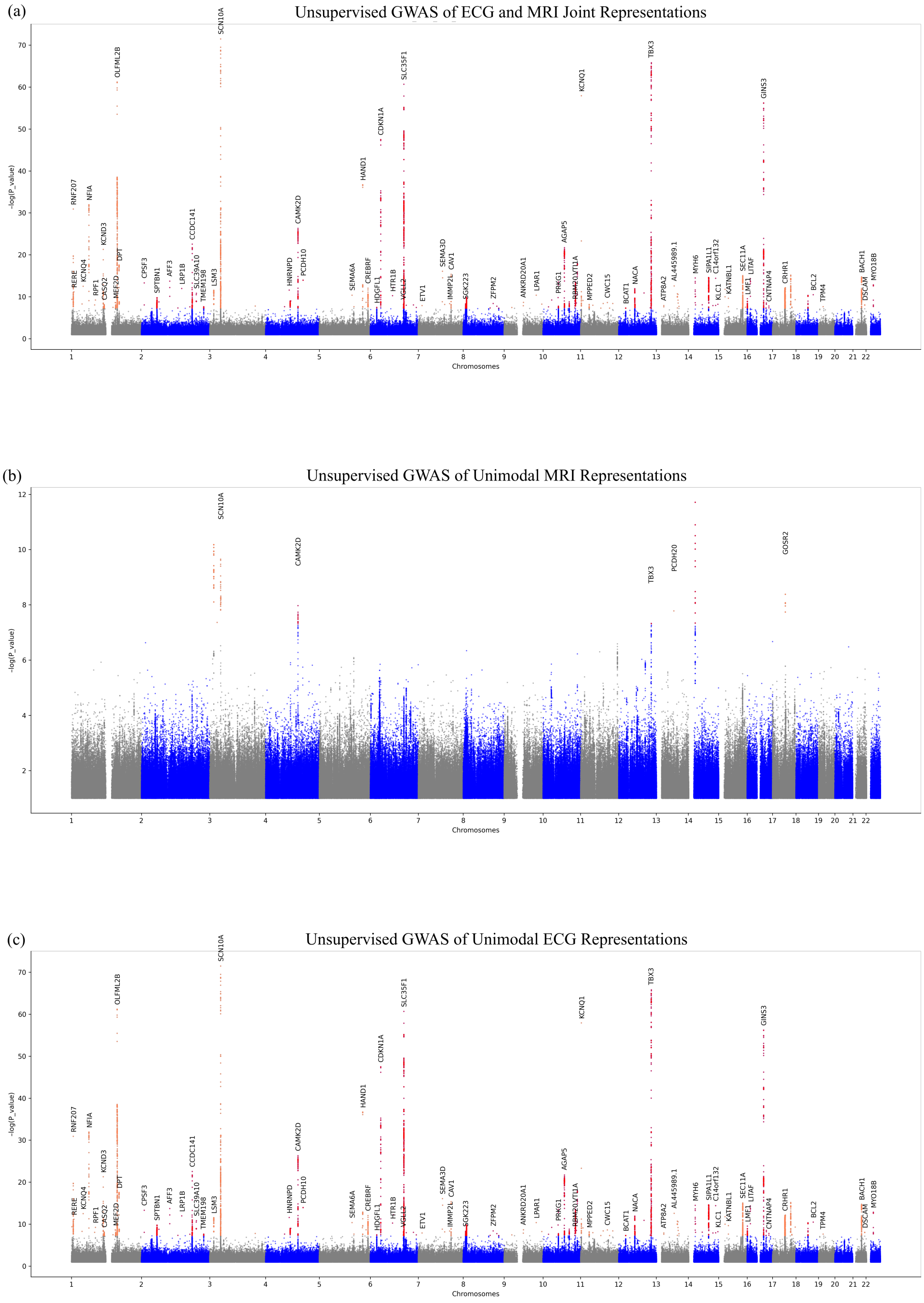

Supplementary Fig. S12: Unsupervised GWAS of cross-modal representations and representations from unimodal autoencoders. (a) Unsupervised GWAS of cross-modal latent representations of both ECG and MRI, i.e., representations corresponding to the instances where both ECG and MRI pairs are available as input to the model. (b) Unsupervised GWAS of MRI representations from a unimodal autoencoder. (c) Unsupervised GWAS of ECG representations from a unimodal autoencoder.

Model	Number of Principal Components of Ancestry	INLP R^2 Threshold	Remaining Latent Dimensionality	Number of Lead SNPs Identified	GC λ
Cross-modal ECG	30	0.001	38	48	1.083
Cross-modal ECG	10	0.002	111	91	1.172
Cross-modal ECG	5	0.002	131	723	1.333
Cross-modal ECG	5	0.01	165	2720	2.72
Unimodal ECG	40	0.001	13	50	1.151
Unimodal ECG	30	0.001	49	97	1.228
Unimodal ECG	20	0.001	85	304	1.338
Cross-modal MRI (256 latent dims.)	10	0.002	136	26	1.086
Cross-modal MRI (512 latent dims.)	30	0.001	202	73	0.984
Unimodal MRI	10	0.002	167	6	1.0

(a)

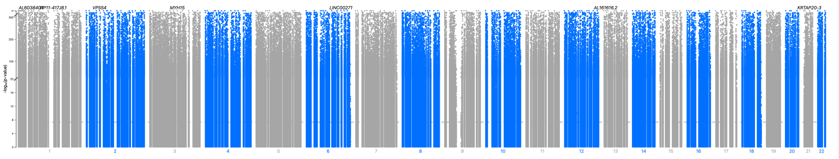

(b)

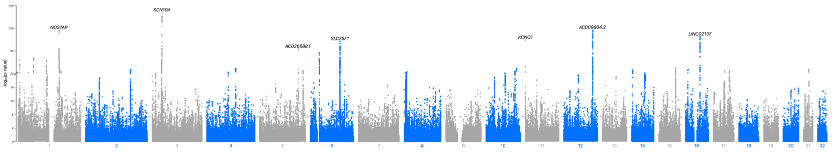

(c)

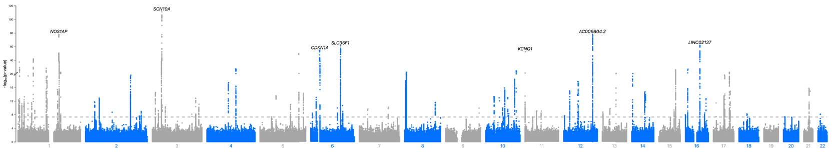

(d)

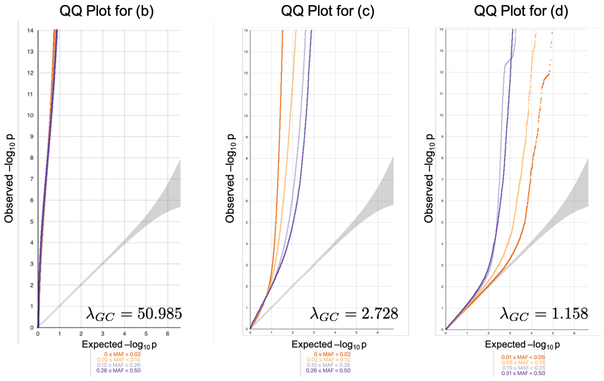

(e)

Supplementary Fig. S13: Impact of varying the number of principal components of ancestry and the threshold for iterative nullspace projection (INLP) on the number of lead SNPs recovered by unsupervised GWAS for cross-modal and unimodal ECG embeddings. (a) Using too few principal components (PCs) of ancestry or using too large of an R^2 threshold yield unsupervised GWAS that are inflated, as is indicated by the λ_{GC} values. (b) Manhattan plot for uncorrected GWAS, which is highly inflated. (c) Manhattan plot for GWAS corrected with 5 PCs and an INLP threshold of 0.01, which is also inflated. (d) Manhattan plot for corrected GWAS with 20 PCs and an INLP threshold of 0.0015, which is no longer inflated. (d) Corresponding QQ plots for the Manhattan plots in (b-d). The λ_{GC} values are large for uncorrected unsupervised GWAS, and decrease to reasonable values after correction.

(a)

Comparison of Lead SNPs Across Embeddings/Phenotypes

	PQ Interval	QRS Duration	QT Interval	QTC Interval	RR Interval
Cross-modal ECG + MRI	0.43	**0.71**	**0.47**	**0.75**	0.39
Cross-modal ECG	**0.53**	0.64	**0.47**	**0.75**	0.44
Cross-modal MRI	0.23	0.32	0.20	0.19	0.33
PQ Interval	1.00	0.29	0.20	0.25	0.33
QRS Duration	0.17	1.00	0.03	0.25	0.06
QT Interval	0.13	0.04	1.00	0.44	**0.78**
QTC Interval	0.09	0.14	0.23	1.00	0.17
RR Interval	0.13	0.04	**0.47**	0.19	1.00
Verweij et al. 2020.	0.45	0.54	**0.47**	0.62	0.44

(b)

Number of Lead SNPs from GWAS

Cross-modal ECG + MRI	Cross-modal ECG	Cross-modal MRI	PQ Interval	QRS Duration	QT Interval	QTC Interval	RR Interval	Verweij et al. 2020.
93	86	33	22	23	18	24	11	72

Supplementary Fig. S14: (a) Unsupervised GWAS of cross-modal representations identifies several lead SNPs associated with the heart and includes those found from GWAS on ECG derived phenotypes and from [2]. Entry (i, j) of the table represents the percentage of lead SNPs identified via GWAS of the phenotype in column j that also arise when performing GWAS of the embedding/phenotype in row i. We observe that lead SNPs identified by unsupervised GWAS of cross-modal representations include several of those from GWAS of PQ interval, QRS duration, QT interval, QTC interval, and RR interval. On the other hand, GWAS based on specific phenotypes (e.g. PQ interval, QRS duration, etc.) identifies lead SNPs that do not overlap much with those from GWAS of other ECG derived phenotypes. Our single unsupervised GWAS of cross-modal ECG representations identifies several of the same lead SNPs as those identified from 500 GWAS of ECG values from [2] upon Bonferroni correction. (b) A count of the number of lead SNPs identified by our unsupervised GWAS compared to GWAS on labelled ECG phenotypes. Our method recovers many more significant SNPs and includes those found via traditional GWAS approaches.

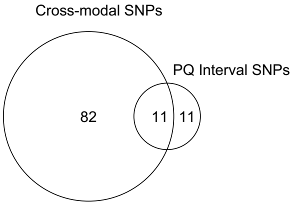

SNP Name	$-\log_{10}$ (p value)
rs561865301	9.59139
rs1482749	9.91033
rs12257568	11.8906
rs4544423	11.895
rs2480714	12.525
rs11148128	7.3769
rs116234738	11.1465
rs7647178	12.0674
rs35712872	10.4323
2:8749400_CCTACT_C	13.8834
rs12117603	8.1044

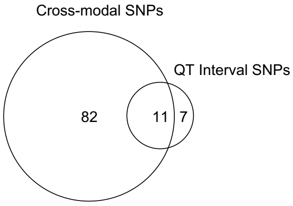

SNP Name	$-\log_{10}$ (p value)
rs1817500	7.84502
rs4811601	7.95739
rs72677052	8.67822
rs758890	16.2342
rs1805128	17.9248
rs9388010	13.6019
rs978202	10.909

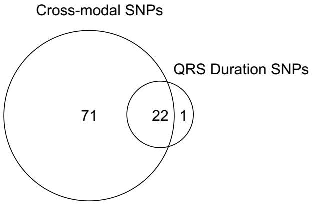

SNP Name	$-\log_{10}$ (p value)
rs7583029	8.79006

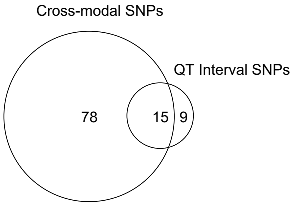

SNP Name	$-\log_{10}$ (p value)
rs62421398	7.81142
rs7017753	7.98811
rs34144937	7.68253
rs3907208	10.4725
rs2349556	13.0059
rs2072413	20.4642
rs1805128	25.2669
17:57463523_AT_A	13.1894
rs79273934	9.00469

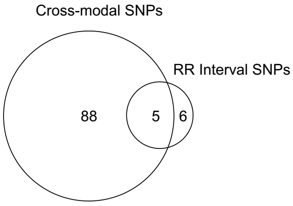

SNP Name	$-\log_{10}$ (p value)
rs60512978	11.8665
rs6701735	7.82161
rs4811602	8.56389
rs17180489	7.88167
rs9388001	14.406
rs78601210	8.67573

Supplementary Fig. S15: Venn diagrams illustrating the difference in SNPs found by the cross-modal unsupervised GWAS and SNPs found by the supervised GWAS on ECG-derived phenotypes. Overall, we observe that the SNPs not found by our method are near the significance cutoff of 5×10^{-8}. Reported p-values are given by two-sided t-tests.

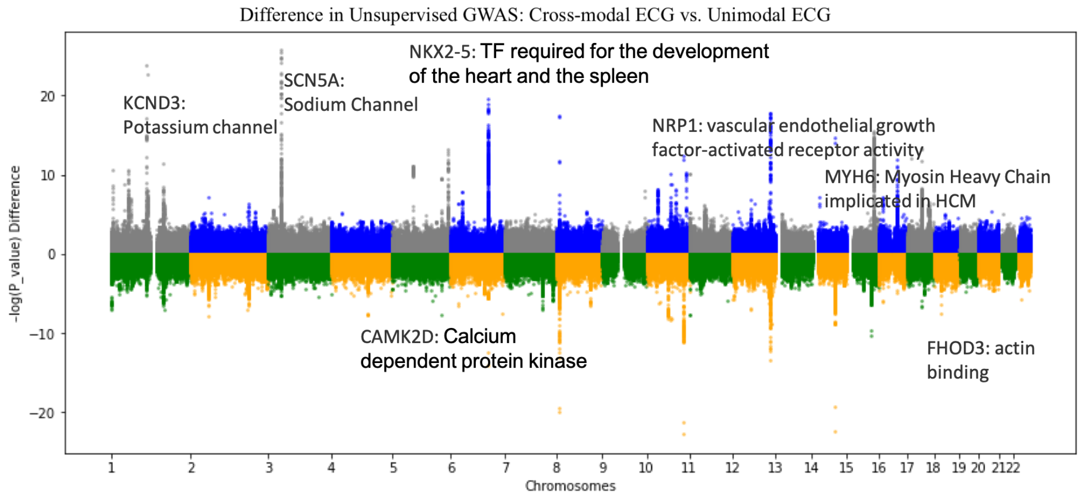

(a)

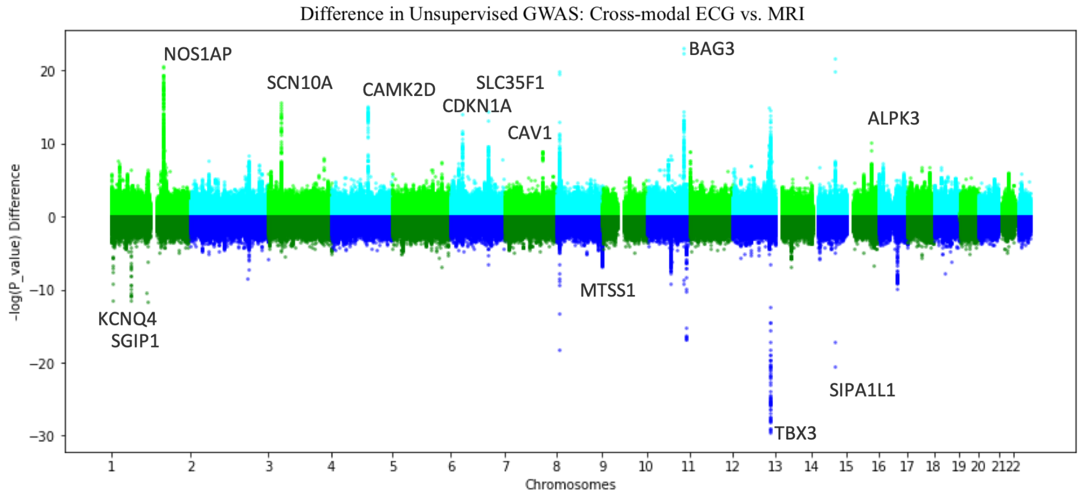

(b)

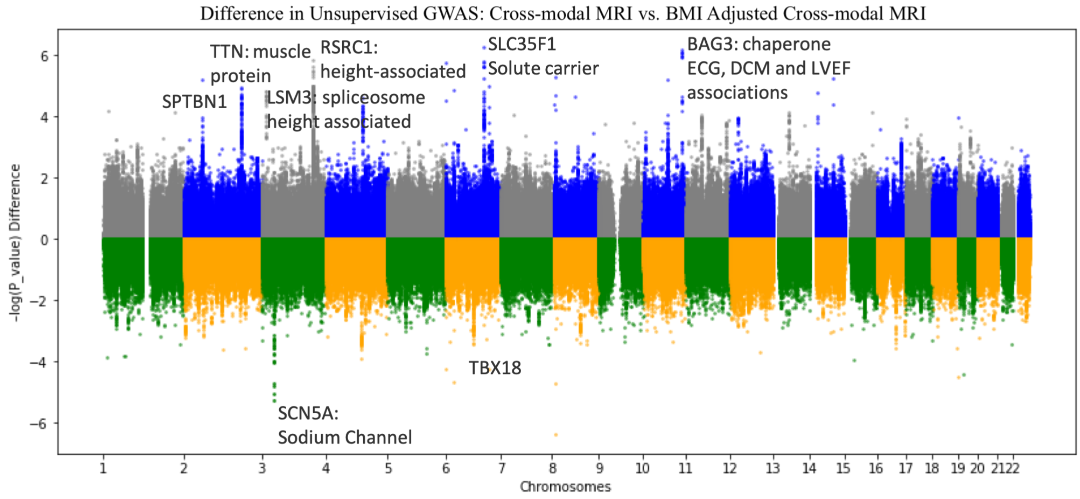

(c)

Supplementary Fig. S16: A visualization of the differences between Manhattan plots resulting from unsupervised GWAS. (a) Cross-modal ECG representations show enriched signals for genes associated with vasculature development, the heart muscle protein, myosin, and ion channels (KCND3, and SCN5A) as compared to unimodal ECG representations. (b) Difference between unsupervised GWAS of cross-modal ECG representations and cross-modal MRI representations shows stronger signals for the cross-modal ECG. (c) BMI-adjustment increases strength of the sodium ion channel SCN5A but also shows reduced significance at sites associated with height.

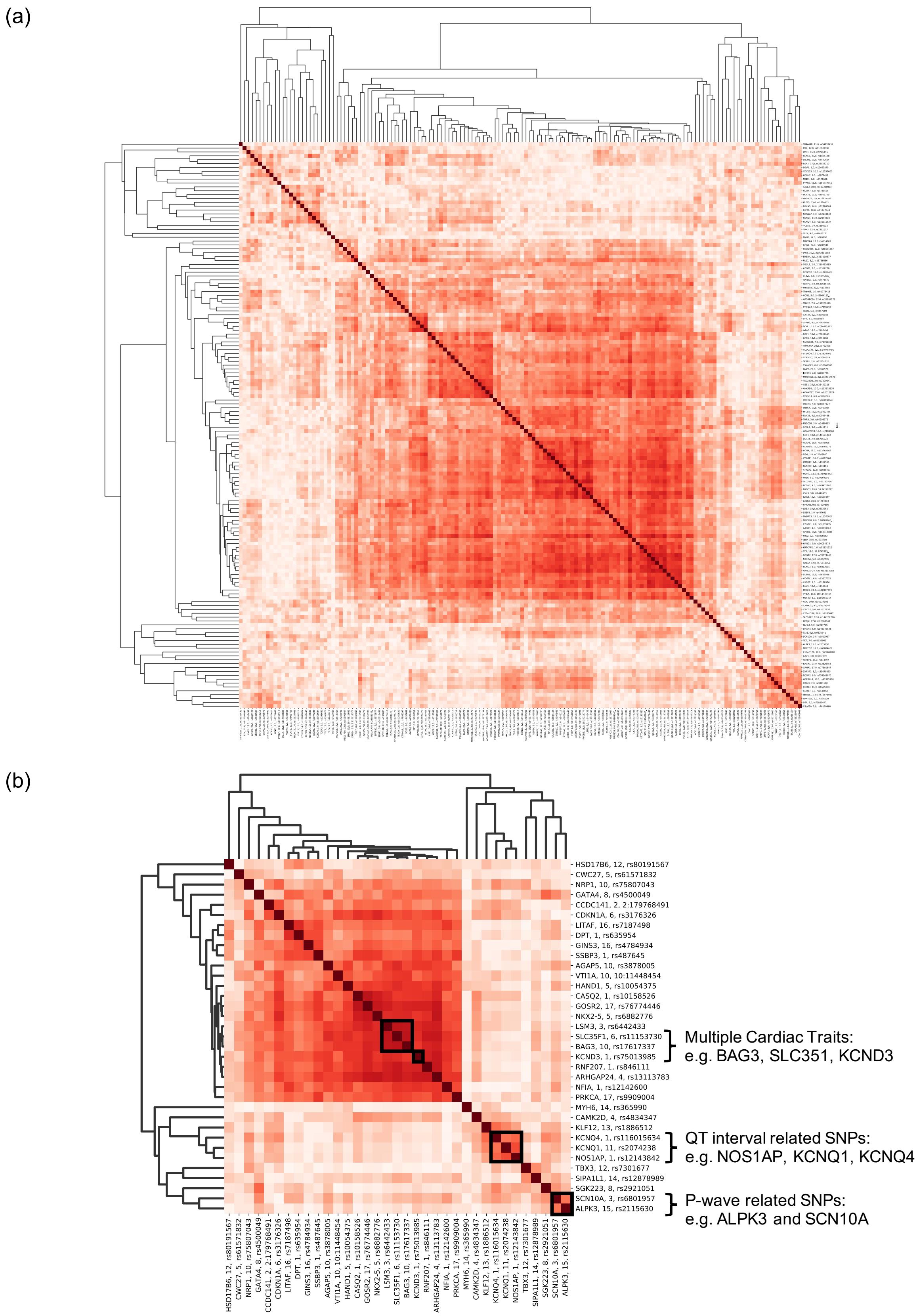

Supplementary Fig. S17: (a) Hierarchical clustering of SNPs by signature, i.e., the vector pointing from the mean embedding of homozygous reference samples to the mean embedding of heterozygous and homozygous alternate samples. Darker colors indicate highly correlated SNP signatures. Several clusters arise including those corresponding to genes associated with the QT interval, genes related to the P-wave, and genes with effects on multiple cardiac traits. (b) Hierarchical clustering on a smaller subset of lead SNPs confirms the robustness of the identified clusters by showing that the SNPs fall into the same phenotypic clusters as in (a).
