# Supplementary material for: Cross-modal autoencoder framework learns holistic representations of cardiovascular state
Source: Nat Commun. 2023 Apr 28;14:2436. doi: 10.1038/s41467-023-38125-0 (PMC10140057; doi:10.1038/s41467-023-38125-0)
Supplement: Supplementary file 4 — Description to Additional Supplementary Information [file 41467_2023_38125_MOESM4_ESM.pdf]

Description of Additional Supplementary Files

Supplementary Video S1: Cardiac MRI videos after decreasing or increasing age by moving in cross-modal latent space.

Supplementary Video S2: Cardiac MRI videos after decreasing or increasing BMI by moving in cross-modal latent space.

Supplementary Video S3: Cardiac MRI videos after decreasing or increasing LVM by moving in cross-modal latent space.

Supplementary Video S4: Cardiac MRI videos after decreasing or increasing RVEDV by moving in cross-modal latent space.

Supplementary Video S5: Cardiac MRI videos after moving in directions corresponding to female or male labeled points in cross-modal latent space.
