# Supplementary material for: Cross-modal autoencoder framework learns holistic representations of cardiovascular state
Source: Nat Commun. 2023 Apr 28;14:2436. doi: 10.1038/s41467-023-38125-0 (PMC10140057; doi:10.1038/s41467-023-38125-0)
Supplement: Supplementary file 10 — Reporting Summary [file 41467_2023_38125_MOESM10_ESM.pdf]

nature portfolio

Corresponding author(s): Caroline Uhler

Last updated by author(s): Dec 7, 2022

Reporting Summary

Nature Portfolio wishes to improve the reproducibility of the work that we publish. This form provides structure for consistency and transparency in reporting. For further information on Nature Portfolio policies, see our Editorial Policies and the Editorial Policy Checklist.

Statistics

For all statistical analyses, confirm that the following items are present in the figure legend, table legend, main text, or Methods section.

n/a	Confirmed	
☐	☒	The exact sample size (*n*) for each experimental group/condition, given as a discrete number and unit of measurement
☐	☒	A statement on whether measurements were taken from distinct samples or whether the same sample was measured repeatedly
☐	☒	The statistical test(s) used AND whether they are one- or two-sided<br>*Only common tests should be described solely by name; describe more complex techniques in the Methods section.*
☐	☒	A description of all covariates tested
☐	☒	A description of any assumptions or corrections, such as tests of normality and adjustment for multiple comparisons
☐	☒	A full description of the statistical parameters including central tendency (e.g. means) or other basic estimates (e.g. regression coefficient) AND variation (e.g. standard deviation) or associated estimates of uncertainty (e.g. confidence intervals)
☐	☒	For null hypothesis testing, the test statistic (e.g. *F*, *t*, *r*) with confidence intervals, effect sizes, degrees of freedom and *P* value noted<br>*Give P values as exact values whenever suitable.*
☒	☐	For Bayesian analysis, information on the choice of priors and Markov chain Monte Carlo settings
☒	☐	For hierarchical and complex designs, identification of the appropriate level for tests and full reporting of outcomes
☒	☐	Estimates of effect sizes (e.g. Cohen's *d*, Pearson's *r*), indicating how they were calculated

Our web collection on statistics for biologists contains articles on many of the points above.

Software and code

Policy information about availability of computer code

Data collection	No software was used for data collection.

Data analysis

For training cross-modal autoencoders:
-python 3.8.10
-numpy 1.22.4
-matplotlib 3.5.2
-tensorflow 2.9.1
-ML4H 0.0.2

For phenotype prediction from cross modal autoencoders:
-python 3.7.11
-pytorch 1.9.11
-cudatoolkit 11.1.74
-pandas 1.3.3
-matplotlib 3.4.2
-numpy 1.21.2
-scikit-learn 0.24.2
-scipy 1.7.1

Link to code: https://github.com/broadinstitute/ml4h

For manuscripts utilizing custom algorithms or software that are central to the research but not yet described in published literature, software must be made available to editors and reviewers. We strongly encourage code deposition in a community repository (e.g. GitHub). See the Nature Portfolio guidelines for submitting code & software for further information.

Data

Policy information about availability of data

All manuscripts must include a data availability statement. This statement should provide the following information, where applicable:

- Accession codes, unique identifiers, or web links for publicly available datasets
- A description of any restrictions on data availability
- For clinical datasets or third party data, please ensure that the statement adheres to our policy

All data use in this study were obtained from UK Biobank. UK Biobank data is available to researchers from research institutions following IRB and UK Biobank application approval. UK Biobank data are available to qualified investigators via application at https://www.ukbiobank.ac.uk. GWAS results can be found in the GWAS catalog https://www.ebi.ac.uk/gwas/studies/, and catalog accession ids are GCST90250896 (for cross-modal ECG and cardiac MRI unsupervised GWAS), GCST90250897 (for cross-modal ECG unsupervised GWAS), and GCST90250898 (for cross-modal cardiac MRI unsupervised GWAS).

Our research complies with all relevant ethical regulations. Access was provided under UK Biobank application #7089. Analysis was approved by the Broad Institute institutional review board.

Human research participants

Policy information about studies involving human research participants and Sex and Gender in Research.

Reporting on sex and gender	Of the samples used for training and evaluating autoencoders that had cardiac MRI available, 21066 individuals were labelled by UK Biobank as genetic sex of male and 23577 individuals were labelled as genetic sex of female.
Population characteristics	N/A
Recruitment	N/A
Ethics oversight	N/A

Note that full information on the approval of the study protocol must also be provided in the manuscript.

Field-specific reporting

Please select the one below that is the best fit for your research. If you are not sure, read the appropriate sections before making your selection.

☒ Life sciences ☐ Behavioural & social sciences ☐ Ecological, evolutionary & environmental sciences

For a reference copy of the document with all sections, see nature.com/documents/nr-reporting-summary-flat.pdf

Life sciences study design

All studies must disclose on these points even when the disclosure is negative.

Sample size

We used all available samples from UK Biobank that contained paired ecg and mri data (38686 samples). We then used a random subset of

Sample size	90% of the data for training and validating cross-modal autoencoders and held out the remaining 10% of data for building models for phenotype prediction.
Data exclusions	We did not exclude any data from the 38686 samples containing ecg and mri pairs from UK Biobank in our analyses.
Replication	We validated that our results can be replicated through analysis of hyper-parameter effects in training cross-modal autoencoders and the use of cross-validation.
Randomization	Training, validation, and test sets were drawn uniformly at random, and we used cross-validation to report means and error bars reflecting one standard deviation.
Blinding	Blinding is not relevant to this study since we are not analyzing effects across different subpopulations or groups.

Reporting for specific materials, systems and methods

We require information from authors about some types of materials, experimental systems and methods used in many studies. Here, indicate whether each material, system or method listed is relevant to your study. If you are not sure if a list item applies to your research, read the appropriate section before selecting a response.

Materials & experimental systems

n/a	Involved in the study
☒	☐ Antibodies
☒	☐ Eukaryotic cell lines
☒	☐ Palaeontology and archaeology
☒	☐ Animals and other organisms
☒	☐ Clinical data
☒	☐ Dual use research of concern

Methods

n/a	Involved in the study
☒	☐ ChIP-seq
☒	☐ Flow cytometry
☒	☐ MRI-based neuroimaging
